# Reciprocity on the Hardwood: Passing Patterns among Professional Basketball Players

**DOI:** 10.1371/journal.pone.0049807

**Published:** 2012-12-07

**Authors:** Robb Willer, Amanda Sharkey, Seth Frey

**Affiliations:** 1 Department of Sociology, University of California at Berkeley, Berkeley, California, United States of America; 2 Booth School of Business, University Of Chicago, Chicago, Illinois, United States of America; 3 Cognitive Science Program, Indiana University, Bloomington, Indiana, United States of America; Universidad Carlos III de Madrid, Spain

## Abstract

Past theory and research view reciprocal resource sharing as a fundamental building block of human societies. Most studies of reciprocity dynamics have focused on trading among individuals in laboratory settings. But if motivations to engage in these patterns of resource sharing are powerful, then we should observe forms of reciprocity even in highly structured group environments in which reciprocity does not clearly serve individual or group interests. To this end, we investigated whether patterns of reciprocity might emerge among teammates in professional basketball games. Using data from logs of National Basketball Association (NBA) games of the 2008–9 season, we estimated a series of conditional logistic regression models to test the impact of different factors on the probability that a given player would assist another player in scoring a basket. Our analysis found evidence for a direct reciprocity effect in which players who had “received” assists in the past tended to subsequently reciprocate their benefactors. Further, this tendency was time-dependent, with the probability of repayment highest soon after receiving an assist and declining as game time passed. We found no evidence for generalized reciprocity – a tendency to “pay forward” assists – and only very limited evidence for indirect reciprocity – a tendency to reward players who had sent others many assists. These findings highlight the power of reciprocity to shape human behavior, even in a setting characterized by extensive planning, division of labor, quick decision-making, and a focus on inter-group competition.

## Introduction

Reciprocity refers to various patterns by which individuals exchange favors, support, goods, or other valued resources. Past research has identified various forms of reciprocity, including direct [1], indirect [2], and generalized [3]. Social scientists have theorized that these forms of reciprocity create patterns of social interaction and provide fundamental building blocks for social institutions [4], [5]. Reciprocity dynamics recur commonly and, have been observed in field settings such as business negotiations [6], the production of open-source software [7], marital partner exchange in indigenous societies [8], and spontaneous truces among soldiers at war [9]. Among nonhuman animals, reciprocity dynamics have been observed in various species, for example direct reciprocity in stickleback fish [10], indirect reciprocity among male song sparrows [11], and generalized reciprocity in rats [12]. Perhaps in part because reciprocity is observed across diverse species, evolutionary theorists have advanced models of how direct [13], indirect [2], and generalized reciprocity [14] could each have emerged as a result of evolutionary processes.

While past research suggests that fundamental motivations lead humans to engage in direct reciprocity, and perhaps also indirect and generalized reciprocity, few studies have explored whether patterns of reciprocity might help explain behavior in highly-structured group settings where resource sharing is largely a product of planning, strategy, and division of labor. However, such organizational contexts are both ubiquitous and socially significant. Further, documenting reciprocity in a setting such as this – where reciprocity is neither readily apparent nor explicit, and where benefits of reciprocity are not clearly evident for either the individual or group – would offer more convincing evidence that engagement in these forms of behavior is in fact based in strong motivations.

To this end, we investigate whether patterns of passing in professional basketball games exhibit the same patterns of reciprocity found with other resource sharing. One might reasonably wonder whether reciprocity plays any role at all in this domain, as passing in professional basketball games is heavily structured as a result of carefully planned strategy and an explicit division of labor on the court prescribing who passes to whom. Further, passing to someone on the basis of past passing patterns, rather than an assessment of what is the most productive pass to make in a given situation, does not clearly benefit either the player or team. Nonetheless, given theory and research suggesting the fundamental nature of reciprocity, it is possible that these dynamics in fact structure passing behavior, producing hidden patterns that would not be immediately observable without systematic analysis. Below we present each form of reciprocity, identifying the type of resource exchange it describes, and highlighting the social psychological mechanisms thought to drive it.

**Figure 1:**
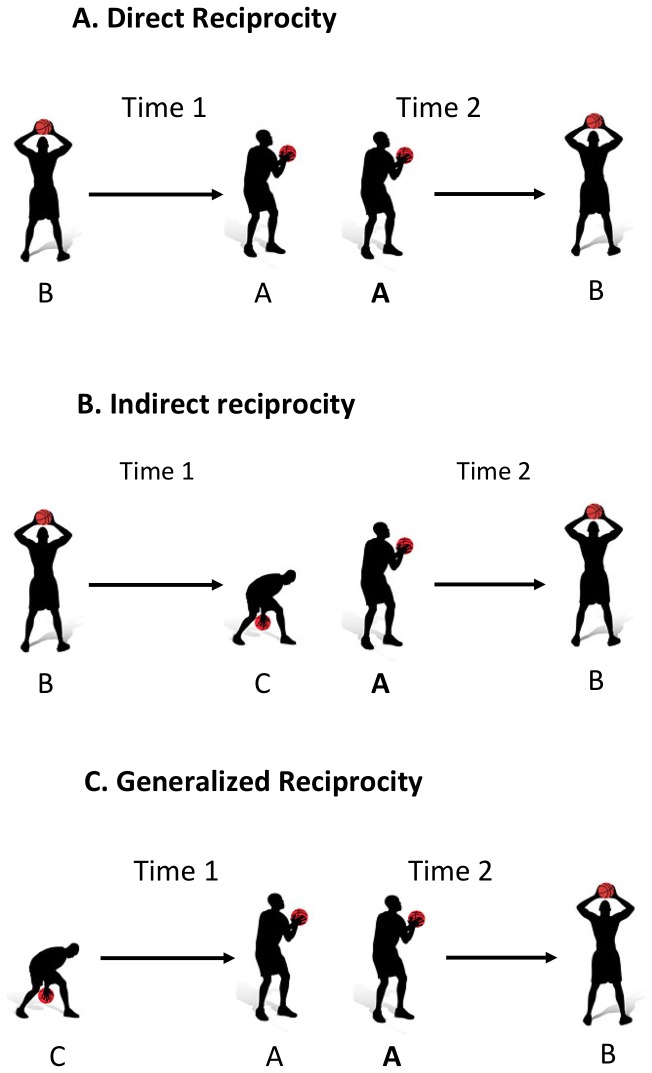
Types of reciprocity in assists. The first panel illustrates direct reciprocity between players A and B. The second panel illustrates indirect reciprocity from focal player A to B, for player B's previous assist to C. The third panel illustrates generalized reciprocity from player A to B, paying forward player C's previous assist to A.

Direct reciprocity involves an actor, A, repaying B for benefits received from him/her in the past. This pattern of reciprocal resource sharing is depicted in Figure 1a. A variety of social psychological mechanisms have been invoked to explain direct reciprocity. Social norms may lead individuals to engage in direct reciprocity because they wish to behave in appropriate ways, fear reputation loss, or wish to avoid social sanctions [1]. The expectation of future interaction with another individual may also stimulate direct reciprocity as a way to build and sustain a mutually beneficial, productive relationship [9]. Finally, the emotional experience of gratitude, felt upon receipt of a favor or gift, can also compel individuals to reciprocate good turns [15].

Indirect reciprocity occurs when a benefactor is rewarded by third parties for behaving generously towards others, i.e., when A rewards B for having given to some third party, C, in the past. This pattern is depicted in Figure 1b. The prospect of indirect reciprocity encourages individuals to behave in generous ways in their social relations, as their prosocial behavior may come to be known and rewarded by other group members [16]. Researchers have argued that generous acts lead individuals to be seen as sincerely motivated to benefit others, a motivation that tends to be respected by others, leading people to preferentially accord status and allocate resources to more generous individuals [17], [18].

Generalized reciprocity involves A repaying benefits to B that A received from some third party, C, in the past. This pattern is portrayed in Figure 1c. In popular vernacular, generalized reciprocity is often referred to as “paying it forward,” a pattern of resource sharing in which generosity is in a sense contagious, with individuals who receive generosity being more likely to behave generously in future interactions. Theorists have argued that the psychology underlying generalized reciprocity may overlap with that underlying direct reciprocity [19]. Individuals who benefit from another person's generosity experience gratitude, and that emotion motivates them to subsequently behave more generously towards third parties [15].

### Empirical overview

Passing in basketball can be viewed as a form of resource sharing and thus might reasonably be subject to the same causal forces shaping exchange in other settings. Thus, in the present study we investigate whether these three fundamental forms of reciprocity identified by past research might help explain patterns of passing in professional basketball games.

Unfortunately total passing data is not available for NBA basketball games. As a result, here we study patterns of assists, passes determined to lead directly to a made basket. Note that this provides an imperfect measure of passing behavior, however there is some reason to think that assists might offer better insight on reciprocity dynamics in this context. One justification of this measure lies in the fact that assists are especially valuable passes, as they lead directly to scoring which benefits the individual and team. Thus, there is reason to think that assists are more likely to be viewed by players as the sort of “valued resources” that forms of reciprocity apply to. As a result, “receiving” assists should feel like receiving valued rewards, more than receiving other passes. In addition, there is good reason to think that players often know that a pass is likely to lead to a scoring opportunity, such as passes to players who are undefended or nearer to the hoop. Thus, it is also likely that benefactors perceive that “giving” assists entails greater value than making other sorts of passes.

For each assist, we know, 1) who has given and who has received the assist, and 2) the exact game time of the event. These data allow us to test the following hypotheses:

*Direct reciprocity hypothesis:* A focal player, A, will be more likely to give an assist to another player, B, if A has received assists from player B in the past.

*Indirect reciprocity hypothesis:* A focal player, A, will be more likely to give an assist to another player, B, if B has given assists to some other players, C, in the past.

*Generalized reciprocity hypothesis:* A focal player, A, will be more likely to give an assist to another player, B, if A has received assists from some other players, C, in the past.

## Materials and Methods

In order to test our hypotheses, we analyzed assists occurring in the 2008–09 NBA season, using data published at http://www.basketballgeek.com and www.dougstats.com. The latter provided season-wide statistics for each player while the former provided play-by-play accounts for more than 1,000 games of the season, including time-stamped records of all assisted baskets. In our reduced dataset, each assist was represented by a set of four player dyads. The dyads included the player who gave the assist, paired with each of the four other players on the floor at the time. A dyad was coded as “1” if an assist occurred between the two players and “0” otherwise. In all, the dataset included 170,756 such dyads. In what follows, we refer to the player giving the assist as “player A” and the potential recipients as “player B.”

We analyzed the data using conditional logistic regression models. Conditional logistic regression models are appropriate for predicting the choice among a set of alternatives as a function of different attributes of the choice set [20]. In this case, we were interested in predicting which player on the floor would be the recipient of a given assist and analyzing whether the choice of a particular player was influenced by reciprocity considerations.

Formally, the model is specified as:
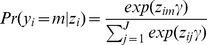
where *y_i_* refers to individual *i's* choice, *m* refers to a particular outcome that could be selected, *z_i_* refers to a set of predictor variables, and *γ* refers to the estimated coefficients associated with each predictor variable.

Coefficients estimated from this model refer to the effect of a unit change in the independent variable on the log odds that player A will choose a particular player B, rather than other potential recipients of an assist.

### Independent variables

#### Test of direct reciprocity

The key independent variable in this analysis was a count of the number of assists A had received from another player, B, but had not yet repaid; i.e., the number of assists A had received from B to that point in the game, minus the number of assists A had given to B. We experimented with different versions of this variable (e.g., a binary measure rather than a continuous metric) but ultimately decided to use the continuous variable because models using this variable fit the data best according to BIC statistics. Because the motivation to reciprocate likely attenuates over time [1], we also interacted the main reciprocity variable with the (logged) number of minutes that player A and player B have been on the floor together since player B last gave A an assist. In cases where player B has never assisted player A, we used the number of minutes that the two have been on the floor together until the current point in the game. We predicted a negative interaction between our indicator of a reciprocation opportunity and this time variable, consistent with the idea that the desire to repay a favor is strongest immediately after receiving something and weakens over time.

#### Test of indirect reciprocity

Indirect reciprocity corresponds to the desire to help someone who has exhibited helping behavior toward others in the past. In this context, if a focal player were motivated by indirect reciprocity, he would be more likely to assist a player who had frequently assisted others, even if that player had not assisted the focal player. Accordingly, we measured drivers of indirect reciprocity with a count of how many assists player B had given to others, not including A. We also interacted this with the (logged) number of minutes player A and player B had been on the floor together since player B last assisted a player other than A. If player B had never assisted anyone or had never assisted anyone other than player A, we included the total amount of time A and B had been on the floor together to that point in the game.

#### Test of generalized reciprocity

Generalized reciprocity represents the idea that a person may be motivated to give to others if he or she has received favors in the past, even if he does not give back directly to those who have given to him or her. To test for generalized reciprocity, we counted how many assists A had received from everyone on the floor, excluding B. Similar to our time-based interaction terms for direct and indirect reciprocity, we included a variable to measure the time that A and B had been on the court together since A last received an assist from anyone besides B.

#### Control variables

We controlled for a variety of factors that might cause a player to be chosen as the recipient of an assist more frequently than others. In order to capture the fact that a player's position is a major driver of the role he plays on the team with respect to assisting behavior, we included indicators for B's position (dummies for center, power forward, small forward and point guard). We also controlled for player B's field goal percentage (shots made per attempt), assists per game, shots attempted per game, and points per game for the 2008–9 season. We included the average number of minutes played per game (logged) by player B. To capture the idea that players who are perceived as having a “hot hand” might tend to receive more assists, we controlled for the number of shots player B had made so far in the current game. To account for the possibility that players might “take turns,” tending to pass to those who had not recently had an opportunity to take a shot, we also controlled for the number of shots player B had attempted in the current game. We also controlled for the number of minutes player A and player B had been on the court together to that point in the current game.

We also controlled for player A's position, average minutes played per game (logged), and average assists per game for the 2008–9 season. Because these characteristics of player A do not vary within a possession, we are unable to estimate their effects by including them in the model as is. As a result, in order to ensure variation on these variables within a given possession, we chose to interact player A's characteristics with the number of minutes A and B have overlapped on the court so far in the current game. Finally, we accounted for possible differences in teams' strategies with respect to assists by including team indicator variables, again interacted with the number of minutes A and B have overlapped on the floor until the current point in the game. Table 1 presents descriptive statistics for the variables used in the analysis.

**Table 1:** Summary Statistics for Variables Used in Analysis of Assist Behavior.

	Mean	Std. Dev.	Min.	Max.
Player B Characteristics*				
Mins. on Court in Current Game with Player A	9.90	7.82	0.02	43.05
Center	0.19	0.39	0.00	1.00
Power Forward	0.23	0.42	0.00	1.00
Small Forward	0.23	0.42	0.00	1.00
Point Guard	0.18	0.38	0.00	1.00
Shooting Guard	0.19	0.39	0.00	1.00
Shots Made/Attempt (Season Avg. %)	0.45	0.59	0.00	1.00
Shots Attempted Per Game (Season Avg.)	11.71	5.65	0.05	25.75
Log Minutes Played Per Game (Season Avg.)	3.27	0.36	0.52	3.68
Assists Per Game (Season Avg.)	7.83	5.20	0.00	32.30
Points Per Game (Season Avg.)	12.06	6.01	0.00	30.20
Shots Made (Current Game)	2.32	2.36	0.00	20.00
Shots Attempted (Current Game)	4.85	4.34	0.00	31.00
Minutes on Court (Current Game)	14.13	9.82	0.03	47.25
Direct Reciprocity Drivers				
Number of Assists Player A Owes to B	0.15	0.46	0.00	7.00
Log Minutes on Court Together Since B Last Assisted A	1.75	1.07	−4.09	3.76
Indirect Reciprocity Drivers				
Number of Assists from B to Anyone Besides A	0.99	1.48	0	18.00
Log Minutes on Court Together Since B Assisted Anyone Besides A	1.34	1.08	−4.09	3.75
Generalized Reciprocity Drivers				
Number of Assists from Anyone Besides B to A	1.00	1.31	0	13.00
Log Minutes on Court Together Since Anyone Besides B Assisted A	1.33	1.07	−4.09	3.70

## Results

Table 2 presents the estimated coefficients from conditional logistic regression models predicting the likelihood of a particular player getting an assist. Model 1 includes control variables. Most control variables operate as expected. Not surprisingly, a player is much more likely to be selected as the recipient of an assist if his field goal percentage is high (*b* = 1.581, *p*<.001). Moreover, the greater a player's average shots attempted per game for the season, the more likely he is to receive an assist (*b* = 0.051, *p*<.001).

**Table 2:** Estimated Coefficients from Conditional Logistic Regression Models Predicting the Recipient of an Assist, Direct Reciprocity.

	(1)	(2)	(3)
Min. on Court in Game w/Player A	−0.006	−0.006	−0.021*
	(0.010)	(0.010)	(0.010)
Center	−0.019	−0.019	−0.020
	(0.023)	(0.023)	(0.023)
Power Forward	0.128***	0.128***	0.127***
	(0.019)	(.019)	(0.019)
Small Forward	0.031	0.031	0.031
	(0.018)	(0.018)	(0.018)
Point Guard	−0.038	−0.038	−0.037
	(0.022)	(0.022)	(0.022)
Shots Made/Attempt (Season Avg. %)	1.581***	1.581***	1.575***
	(0.160)	(0.160)	(0.160)
Shots Attempted Per Game (Season Avg.)	0.051***	0.051***	0.051***
	(0.004)	(0.004)	(0.004)
Log Min. Played Per Game (Season Avg.)	−0.224***	−0.224***	−0.238***
	(0.032)	(0.032)	(0.033)
Assists Per Game (Season Avg.)	−0.013***	−0.014***	−0.013***
	(0.002)	(0.002)	(0.002)
Points Per Game (Season Avg.)	−0.002	−0.002	−0.002
	(0.003)	(0.003)	(0.003)
Shots Made in Current Game	0.001	0.001	0.001
	(0.005)	(0.005)	(0.005)
Shots Attempted in Current Game	−0.000	−0.000	0.001
	(0.004)	(0.004)	(0.004)
Minutes on Court in Current Game	−0.007***	−0.007***	−0.007***
	(0.002)	(0.002)	(0.002)
(1) Number of Assists Player A owes B		0.001	0.101***
		(0.013)	(0.027)
(2) Log Minutes on Court Together			0.075***
Since B Last Assisted A			(0.014)
(1) X (2)			−0.035**
			(0.013)
Wald Chi-Squared	1746***	1747***	1779***
Degrees of Freedom	48	49	51

Note: Robust Standard Errors Clustered on Assists. All models include controls for team and Player A factors, as described in Data & Methods section (results are not reported for brevity). *N* = 170,756 Player A-Player B Dyads;

* *p*<.05,

** *p*<.01,

*** *p*<.001. All tests two-tailed.

In model 2, we tested for direct reciprocity by including the count of how many assists player A “owes” player B. This variable is not significant. However, the results of model 3 provide evidence of a direct reciprocity effect once we account for the fact that the motivation to reciprocate is likely to decline over time. Model 3 includes the interaction of the count of assists owed and the (logged) time since B last assisted A. Conditional on giving an assist to anyone, for each additional assist received from B that has not yet been repaid, odds are 10.6% (e^0.101^−1) higher that player A will assist player B. The negative interaction term indicates that this effect diminishes over time, consistent with our expectation.

Table 3 presents results of tests for indirect and generalized reciprocity. In model 1, we added a variable that captures the total number of assists player B has given to others besides A. This term was not significantly related to A's likelihood of assisting B in this model. Model 2 includes the interaction of the count of assists given by B to others besides A and the (logged) number of minutes since player B last assisted someone besides A. In this model the time since B assisted someone else and the count of assists by B were both positively related to A's likelihood of assisting B, however the interaction of these terms was not. Given that the effect of B's past assisting behavior to others besides A only affected A's likelihood of assisting B in this latter model, and the effect did not interact with the time since the last assist B had given to another teammate as would be expected, we conclude that these results do not support the existence of indirect reciprocity in this setting.

**Table 3:** Estimated Coefficients from Conditional Logistic Regression Models Predicting the Recipient of an Assist, Indirect and Generalized Reciprocity.

	(1)	(2)	(3)	(4)
Min. on Court in Game w/Player A	−0.006	−0.009	−0.006	−0.013
	(0.010)	(0.010)	(0.010)	(0.010)
Center	−0.019	−0.020	−0.019	−0.018
	(0.023)	(0.023)	(0.023)	(0.023)
Power Forward	0.128***	0.127***	0.128***	0.128***
	(0.019)	(0.019)	(0.019)	(0.019)
Small Forward	0.031	0.031	0.031	0.031
	(0.018)	(0.018)	(0.018)	(0.018)
Point Guard	−0.038	−0.038	−0.038	−0.038
	(0.022)	(0.022)	(0.022)	(0.022)
Shots Made/Attempt (Season Avg. %)	1.578***	1.571***	1.579***	1.578***
	(0.160)	(0.160)	(0.160)	(0.160)
Shots Attempted Per Game (Season Avg.)	0.051***	0.051***	0.051***	0.051***
	(0.004)	(0.004)	(0.004)	(0.004)
Log Min. Played Per Game (Season Avg.)	−0.222***	−0.226***	−0.224***	−0.227***
	(0.032)	(0.033)	(0.032)	(0.032)
Assists Per Game (Season Avg.)	−0.014***	−0.014***	−0.014***	−0.014***
	(0.002)	(0.002)	(0.002)	(0.002)
Points Per Game (Season Avg.)	−0.002	−0.002	−0.002	−0.002
	(0.003)	(0.003)	(0.003)	(0.003)
Shots Made in Current Game	0.002	0.002	0.001	0.001
	(0.005)	(0.005)	(0.005)	(0.005)
Shots Attempted in Current Game	−0.000	−0.000	−0.000	0.000
	(0.004)	(0.004)	(0.004)	(0.004)
Minutes on Court in Current Game	−0.008***	−0.008***	−0.007***	−0.008***
	(0.002)	(0.002)	(0.002)	(0.002)
(3) Number of Assists from B to	0.006	0.018*		
Anyone Besides A	(0.005)	(0.007)		
(4) Log Minutes on Court Together		0.022*		
Since B Last Assisted A		(0.009)		
(3) X (4)		−0.005		
		(0.004)		
(5) Number of Assists from Anyone			−0.014	−0.022
Besides B to A			(0.012)	(0.034)
(6) Log Minutes on Court Together				0.038**
Since Anyone Besides B Assisted A				(0.012)
(5) X (6)				0.005
				(0.006)
Wald Chi-Squared	1747***	1753***	1748***	1767***
Degrees of Freedom	49	51	49	51

Note: Robust Standard Errors Clustered on Assists. All models include controls for team and Player A factors, as described in Data & Methods section (results are not reported for brevity). *N* = 170,756 Player A-Player B Dyads;

* *p*<.05,

** *p*<.01,

*** *p*<.001. All tests two-tailed.

Models 3 and 4 of Table 3 test for generalized reciprocity by including the number of assists player A has received from anyone besides player B. We interacted this variable with the time player A and B have been on the court together since A last received an assist from someone other than B. Neither of these terms was significant. Note, however, that it would be difficult for us to find strong evidence for both direct and generalized reciprocity using our analytic approach in this setting. It is possible that both assists earned from B in the past and players besides B could both additively contribute to A's likelihood of assisting B, relative to other teammates (the predictions of our direct and generalized reciprocity hypotheses). However, the stronger A's motivation is to engage in direct reciprocity, the less likely we would be to observe that A will select B from among his teammates to assist after receiving assists from teammates besides B, since a strong direct reciprocity motivation would lead A to reciprocate those other teammates directly.

## Discussion

Overall, the results of our analyses suggest that reciprocity is responsible for some passing behavior among NBA players. We found evidence for direct reciprocity as a factor in the choice of whom a player was likely to assist. Individuals were more likely to assist another player who had assisted them in the past. Further, this effect was strongest soon after the original assist. The effect of having received an assist on the likelihood of reciprocation was greatest immediately after an assist was received and diminished as time passed from the receipt of the benefit, consistent with reciprocity dynamics in other settings.

Indirect and generalized reciprocity, on the other hand, did not seem to influence assist behavior. The lack of consistent evidence for indirect reciprocity is perhaps not surprising. Assisting others may often be seen as an expected behavior in this context, especially among those players responsible for setting up the team's offense, like guards (who are responsible for the greatest number of assists). Thus, being responsible for an assist may not be seen as a strong indicator that one is generous and deserves to be rewarded by third parties. Still, given the robustness of past research on indirect reciprocity, the prospect that more generous basketball players are subsequently rewarded by their teammates – even those they did not directly benefit – deserves further attention. The lack of evidence for generalized reciprocity may be a product of the subtlety of this effect. While past research has documented tendencies for people to “pay forward” favors received, these effects appear to be much smaller than corresponding direct reciprocity effects.

In the setting we studied, individuals tended to repay assists received from teammates with direct reciprocity, though neither individual nor team performance was clearly served by such behavior. But while testimony to the power of reciprocity, our findings cannot speak to what psychological mechanism(s) – e.g., internalization of cultural norms, feelings of indebtedness, a hope that reciprocity might lead to future benefits for oneself – might drive these effects, providing a potentially fruitful avenue for future investigation. These findings underscore the strength of human motivations to engage in direct reciprocity, demonstrating that it obtains even in a setting where individual performance is highly salient and rewarded, player roles are clearly defined, and within-game strategy and coaching prescribes much passing behavior.
